# LncRNA GAS5 suppresses ovarian cancer by inducing inflammasome formation

**DOI:** 10.1042/BSR20171150

**Published:** 2018-03-16

**Authors:** Jie Li, Chen Yang, Yinguang Li, Aiyue Chen, Li Li, Zeshan You

**Affiliations:** 1 Department of Gynecology, The Eastern Hospital of the First Affiliated Hospital of Sun Yat-sen University, Guangzhou, Guangdong 510700, China; 2 Gynecology Department of Physical Examination Center, The Eastern Hospital of the First Affiliated Hospital of Sun Yat-sen University, Guangzhou, Guangdong 510700, China; 3 Department of Gynecology, The First Affiliated Hospital of Sun Yat-sen University, Guangdong 510080, P.R. China

**Keywords:** apoptosis, cell proliferation, growth arrest-specific 5, inflammasome, long non-coding RNA, ovarian cancer

## Abstract

Objective: Long noncoding RNA growth arrest-specific transcript 5 (lncRNA GAS5) is involved in various kinds of cancer. However, the role of lncGAS5 in the development of ovarian cancer remains unclear. In the present study, we explored the cellular mechanism and clinical value of lncRNA GAS5 in ovarian cancer.

Methods: Quantitative real-time PCR was used to detect mRNA level of lncRNA GAS5 in 20 ovarian cancer tissues. The effect of lncRNA GAS5 on cell proliferation was performed using CCK-8 assay. Cell apoptosis was evaluated by flow cytometry. Western blotting was used to detect the protein level of lncRNA GAS5 potential target. Standard sandwich ELISA was used to quantify the level of inflammatory cytokines. The cells with stable expression of lncRNA GAS5 were injected into nude mice to study the effect of lncRNA GAS5 on tumorigenesis *in vivo.*

Results: The expression of lncRNA GAS5 was significantly decreased in ovarian cancer tissues. Decrease in lncRNA GAS5 expression resulted in increased cell proliferation and colony formation and reduced ovarian cancer cell apoptosis. In contrast, exogenous overexpression of lncRNA GAS5 in ovarian cancer cells inhibited proliferation, colony formation, and apoptosis in ovarian cancer cells. In addition, the role of lncRNA GAS5 in ovarian cancer was associated with inflammasome formation and pyroptosis.

Conclusion: These results suggested that lncRNA GAS5 acts as tumor suppressor and could be used as a potential treatment target for diagnosis and therapy of ovarian cancer.

## Introduction

Ovarian cancer is the fifth most common cancer type in females worldwide, and the second most frequent malignant tumor in females, which accounts for almost 3% of cancer in females [1]. Several hereditary and environment factors have been found to contribute to the development of ovarian cancer. For example, estrogens play important roles in cancer development, increasing cell proliferation, and enhancing cancer cell invasion capacity [2]. While progress has been made in understanding the pathogenesis of ovarian cancer, effective strategies are still short of lowering the incidence and death rate associated with ovarian cancer. Thus, to explore mechanism of carcinogenesis in ovarian cancer is critical to develop new treatment methods for clinical application.

Studies have suggested that over 98% of the human genome contains noncoding proteins sequences, with a majority of them being transcribed into nonprotein-coding RNA transcripts [3]. The size of these transcripts differs from very small RNAs, defined as microRNAs (miRNAs), which contains 20–25 base pairs, to long noncoding RNAs (lncRNAs) that contain over 100 kb [4]. LncRNAs are capable of interacting with different types of biomolecules, such as DNA, RNA and proteins, and play vital roles in gene expression regulation at transcriptional, post-transcriptional, and epigenetic levels [5]. LncRNAs play a critical role in tumorigenesis and tumor progression [6]. LncRNAs can be divided into three types: well-studied tumor-suppressor genes (including LncRNA antisense noncoding RNA in the INK4 locus (ANRIL) and lncRNA maternally expressed gene 3 (MEG3)) [7,8], oncogenes, tumor-suppressor genes, and duplex lncRNAs according to their functions [9]. LncRNA Hox transcript antisense intergenic RNA (HOTAIR) is widely studied as an oncogenic lncRNA that plays an important role in tumor pathogenesis. Up-regulated LncRNA HOTAIR expression acts as a poor prognostic biomarker of patient outcomes with tumor metastasis [10,11]. Nevertheless, limited researches study the role of lncRNAs in ovarian cancer.

Accumulated evidence have shown that chronic inflammation could lead to tumorigenesis, if malignant cells suppress immunologic surveillance [12]. Thus, carcinogenesis and tumor progression are either stimulated or restrained by inflammatory and immune processes respectively. Inflammatory processes are derived from formation of multiprotein complexes such as inflammasomes [13] and from pyroptosis, a process of programmed cell death, but significantly distinct from apoptosis [14]. Inflammasome, a protein complex formed by ASC protein and caspase-1 through its CARD domain, plays a promotive role in activation of caspase-1. Then, proforms of inflammatory cytokines such as IL-18 and IL-1β are activated by cleaved caspase 1 [15]. Inflammasomes can also take part in tissue homeostasis, inflammation, and immune response that interfere with the formation, progression, and therapeutic response of cancer.

In the present study, we investigated the clinical implication and functions of abnormal expression of lncRNAs in ovarian carcinogenesis. LncRNA growth arrest-specific transcript 5 (GAS5) was found to be consistently down-regulated and acted as a tumor suppress gene in various cancers, such as gastric cancer, prostate, and breast cancer [16–18]. However, limited information is available on the role and function of lncRNA GAS5 in ovarian cancer. Thus, we attempted to detect the expression of GAS5 in clinical ovarian cancer sample and investigated the effect of altered expression of GAS5 on ovarian cancer cells. Our findings showed that loss of lncRNA GAS5 in ovarian cancer cells may contribute to the tumor progression and could be a potential therapeutic target for diagnosis and therapy for clinical application.

## Materials and methods

### Clinical specimens

Paired samples of ovarian tissues and corresponding noncancerous ovarian tissues were obtained from 20 patients who underwent surgery at the Eastern Hospital of the First Affiliated Hospital of Sun Yat-sen University, China. The tissues were stored in frozen liquid nitrogen at −80˚C until use. The present study was approved by the Research Ethics Committee of the Eastern Hospital of the First Affiliated Hospital of Sun Yat-sen University, China. All the study protocols dealing with patients conformed to the ethical guidelines of the Helsinki Declaration. Informed consent was informed from each participant prior to experiments.

### Cell lines and culture conditions

Human ovarian surface epithelial cells (IOSE25) and four human ovarian cancer cell lines (SKOV3, OVCAR-3, A2780, and 3AO) were obtained from the American Type Culture Collection (ATCC) (Manassas, VA, U.S.A.). The cell lines were maintained according to the vendor’s instructions. SKOV3 cells were cultured in McCoy’s 5A medium (Sigma) with 10% fetal bovine serum (FBS) (Gibco, Carlsbad, CA, U.S.A.). OVCAR-3 cells were cultured in RPMI-1640 medium (Sigma) with 20% FBS. A2780 and 3AO cell lines were cultured in Dulbecco’s modified Eagle’s medium (DMEM) with 10% FBS. All the media contained 1% penicillin–streptomycin (100 U/ml penicillin and 100 µg/ml streptomycin). All the cell lines were cultured and maintained in a humidified incubator at 37˚C and supplemented with 5% CO_2_.

### RNA extraction and quantitative real-time PCR (qRT-PCR) analysis

TRIzol reagent (Invitrogen, Massachusetts, U.S.A.) was used to extract total RNA from clinical tissue samples or cultured cells. Two micrograms of total RNA was transcribed into cDNA according the manufacture’s protocol (Takara, China). Quantitative real-time PCR analysis was performed using SYBR-Green Real-Time Master Mix (Takara, China). The PCR primers for GAS5 were: forward 5′-TGGTTCTGCTCCTGGTAACG-3′, reverse 5′-AGGATAACAGGTCTGCCTGC-3′; for GAPDH: forward 5′-GTCAAGGCTGAGAACGGGAA-3′, reverse 5′-AAATGAGCCCCAGCCTTCTC-3′. qRT-PCR and data collection were performed using Applied Biosystem Viia 7 Real Time PCR system (ABI, U.S.A.). The relative expression of GAS5 was analyzed and normalized using the 2^−∆∆*C*^_t_ method relative to GAPDH.

### Protein extraction and Western blotting

Total proteins were extracted from cultured cells with RIPA buffer, and protein concentration was determined using BCA protein assay kit (Thermo Fisher Scientific). Equal amounts of proteins were separated using SDS/10% polyacrylamide gel electrophoresis (SDS/PAGE), transferred to 0.22 µm polyvinylidene difluoride (PVDF) membranes, and incubated with specific antibodies. Proteins were detected using chemiluminescence (ECL). The intensity of the bands was quantified by ImageJ. Primary antibodies, ASC (ab227502, ABCAM), caspase-1 (ab62698, ABCAM), p-caspase-1, IL-1β (BA3711, BosterBio), p-IL-1β, IL-18 (PB0057, BosterBio), p-IL-18, and GAPDH (A00227-1, BosterBio) were obtained from Cell Signaling Co. (Cell Signaling Technology, U.S.A.).

### Overexpression and knockdown expression of GAS5

In order to produce a GAS5 expression vector, the entire sequence of human GAS5 (2,651 bp) was synthesized and cloned into the pCDNA3.1 vector. The cloned plasmids were double confirmed by DNA sequencing (Sangon, Shanghai, China). The cloned plasmid with pCDNA3.1-GAS5 (2 μg) or the empty vector (2 μg) was transfected into the cells using Lipofectamine 2000 (Invitrogen) in serum-free medium according to the manufacturer’s instructions. siRNA (5′-UCUUCAAUCAUGAAUUCUGAG-3′) targeting GAS5 was used to knockdown the expression of GAS5. Nontargeting sequence (5′-ACGUGACACGUUCGGAGAATT-3′) was used as a negative control.

### Colony formation

The cells were seeded at about 1000 cells per well of plate and cultured for 14 days to allow colony formation. Crystal Violet (0.1%) in 50% methanol and 10% glacial acetic acid was used to stain the colonies in order to quantify the number of colonies.

### Cell apoptosis assay

Cell apoptosis was determined by cytometry using Annexin V-FITC/propidium iodide (PI) apoptosis detection kits (LiankeBio, Zhejiang, China) according to the manufacturer’s instructions. In brief, the cells were harvested and washed with cold phosphate-buffered saline (PBS). The cells were resuspended and stained using the Annexin V-FITC/PI apoptosis detection kit in accordance with the manufacturer’s instructions. The samples were analyzed using Becton-Dickinson flow cytometer. Annexin V(+)/PI(-) and Annexin V(+)/PI(+) represent the ovarian cancer cells in early and late apoptosis/necrosis stage respectively.

### Cell cycle distribution assay

Cell cycle distribution was determined using flow cytometry. In brief, the cells transfected with siRNA or NC were incubated for 48 h. The cells were collected and placed in flow tube after washing for three times with PBS. One milliliter of 70% ethanol was added to flow tube for cell resuspension with soft stirring. The cells were stained with 300 µl of propidium iodide (PI), and then cell cycle distribution measurements were conducted using Flow Cytometer (BD, FACSCalibur).

### Cell proliferation test

CCK8 kit (Lianke Bio, China) and EDU assay kit (RIBOBIO, China) were used to determine cell proliferation of ovarian cancer cell. CCK8 assay: 10^5^ cells were plated in 96-well plate after transfection with siRNA or NC. The cells were transferred into an incubator for 24, 48, and 72 h. Finally, CCK8 working reagent was added to the wells and incubated for 2 h. Culture solution was read at 450 nm absorbance.

### ELISA and LDH release assay

ELISA assay was used to detect the level of IL-1β in human ovarian cancer cell lines by human IL-1β pre-coated ELISA kit (R&D, Shanghai, China). LDH release assay was performed using LDH release kit (Beyotime Biotechnology, Beijing, China) in accordance with the manufacture’s instruction. Data were collected using microplate reader (Thermo, Massachusetts, U.S.A.).

### *In vivo* tumor growth assay

BALB/c mice were obtained from animal experimental center of Sun Yat-sen University, and animal experiments were approved by Ethic Committee of Animal Experiments of The Eastern Hospital of the First Affiliated Hospital of Sun Yat-sen University. Experiments were conducted according to our previous study [19]. The mice were randomly divided into three groups for PcDNA3.1-GAS5 or pcDNA3.1 vector stably transfected SKOV3 cells were harvested and washed with PBS, then resuspended at a concentration of 5 × 10^7^ cells/ml for injection. A volume of 100 μl of the suspended cells was subcutaneously injected into the right side of the posterior flank of each mouse. Meanwhile, inflammasome inhibitor Yvad-CMK was injected into tumor in GAS5 overexpressed cell. The subcutaneous tumor growth was examined every 5 days. At 40 days’ post injection, the mice were killed after anesthesia, and tumor were obtained to weight.

### Statistical analysis

Statistical analysis was performed using SPSS 21.0. Statistical significance between groups was analyzed using student’s *t*-test or a one-way ANOVA, followed by Turkey test. *P* value of less than 0.05 was considered statistically significant. All the experiments were performed three times.

## Results

### Expression of lncRNA GAS5 in human ovarian cancer tissue

**Figure 1:**
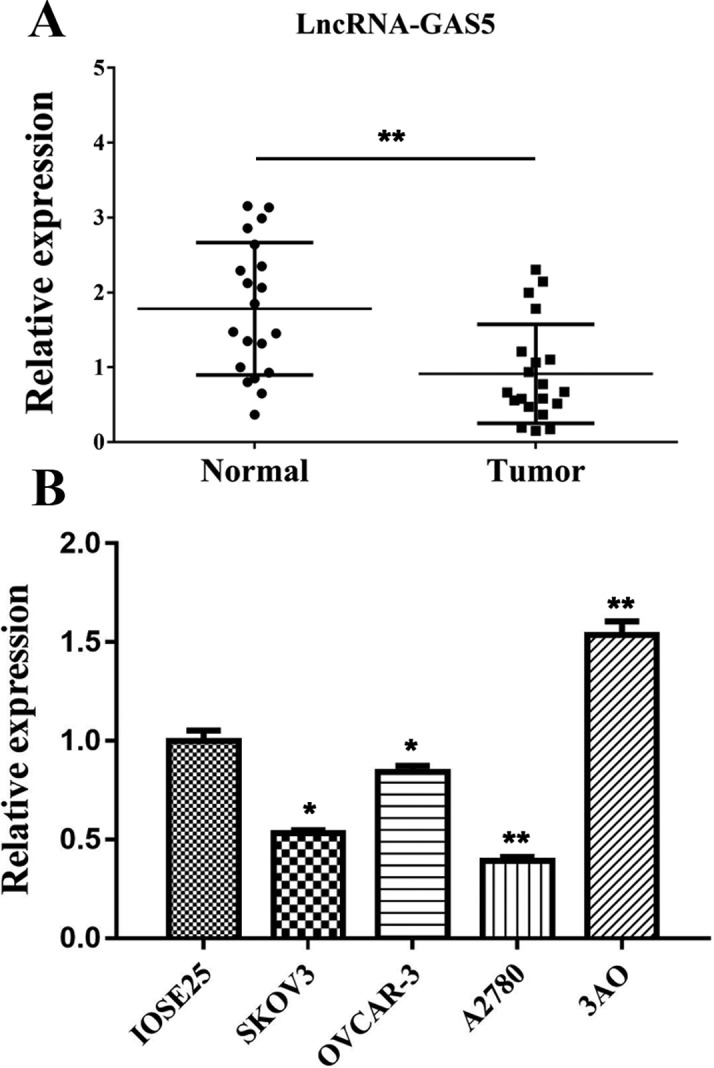
Expression of lncRNA GAS5 in clinical tissues and cell lines (**A**) Expression of GAS5 in normal (paracarcinoma tissue) and tumor tissues. (**B**) Expression of GAS5 in human ovarian surface epithelial cells, IOSE25 and ovarian cancer cell lines, SKOV3, OVCAR-3, A2780, and 3AO; *indicates *P*<0.05, **indicates *P*<0.01.

In order to investigate the expression level of lncGAS5 in human ovarian cancer tissue, 20 clinical patients were recruited for the study. Both of the excised ovarian cancer tissues and matched adjacent normal tissues were collected for the analysis. qRT-PCR was used to examine lncGAS5 expression in the included samples. As demonstrated in Figure 1A, the expression level of GAS5 was significantly down-regulated in ovarian cancer tissues as compared with the corresponding adjacent normal tissues.

To investigate the effect of GAS5 on the malignant behavior of these ovarian cancer cells, we tried to define whether loss of GAS5 expression contributed to the ovarian cancer progression. Five ovarian cancer cell lines SKOV3, OVCAR-3, A2780, 3AO, and human ovarian epithelium cells (IOSE25) were used to detect the relative expression of lncRNA GAS5 (Figure 1B) using qRT-CPR. Finally, 3AO and A2780 cell lines presenting overexpressed and lowest expression of GAS5 respectively among cell lines were selected for further exploration.

### LncRNA GAS5 depletion promotes viability of ovarian cancer cells

**Figure 2:**
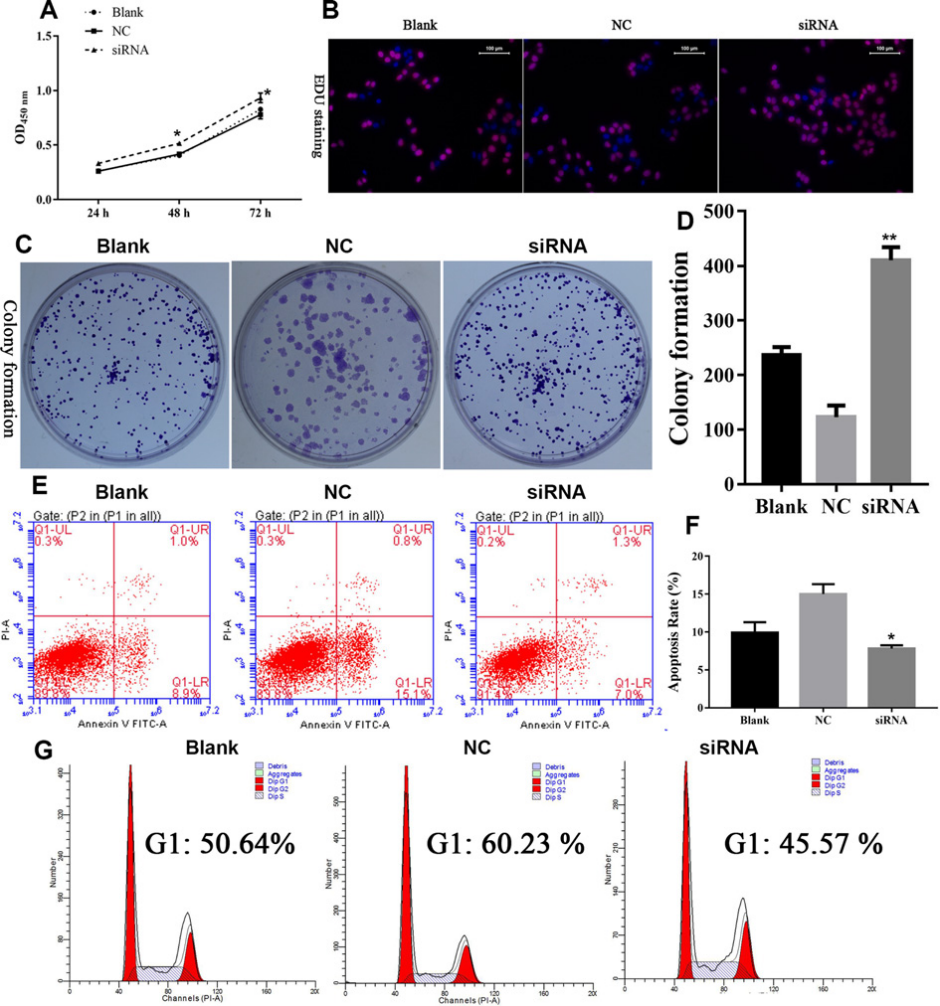
Knockdown of lncRNA GAS5 promotes cancer malignant behavior in 3AO cell line (**A**) Transfection of siRNA–GAS5 induced cell proliferation. (**B**) siRNA induced EdU staining. (**C** and **D**) Colony formation significantly elevated post transfection with siRNA–GAS5. (**E** and **F**) Relatively lower apoptosis rate was detected in siRNA transfected cells. (**G**) G1 phase block was induced after siRNA–GAS5 transfected into cells; *indicates *P*<0.05, ** indicates *P*<0.01.

To explore the effect of GAS5 expression on cell proliferation, CCK8 assay, EDU staining, and colony formation assay were conducted following transfection of 3AO cells with siRNA. As shown in Figure 2A, higher cell viability rate was observed in siRNA group as compared with NC group. In addition, EdU signal intensity was increased in siRNA–GAS5 group for 48 h after knockdown of lncRNA GAS5 expression (Figure 2B). Further, colony formation was also enhanced in shGAS5 group (Figure 2C and D).

To investigate the rate of cell apoptosis and cell cycle distribution after GAS5 depletion, flow cytometry was used. As shown in Figure 2E, cell apoptosis was significantly decreased after cells were transfected with lncRNA GAS5 knockdown as compared with cells transfected with NC (negative control) (Figure 2E and F). Additionally, cell cycle distribution assay showed significantly decreased percentage of cells in G1 phase with lncRNA GAS5 knockdown (Figure 2G).

These results indicated that GAS5 depletion increases cell division, reduces cell apoptosis, and enhances cell proliferation.

### LncRNA GAS5 depletion inhibits inflammasome formation and pyroptosis *in vitro*

**Figure 3:**
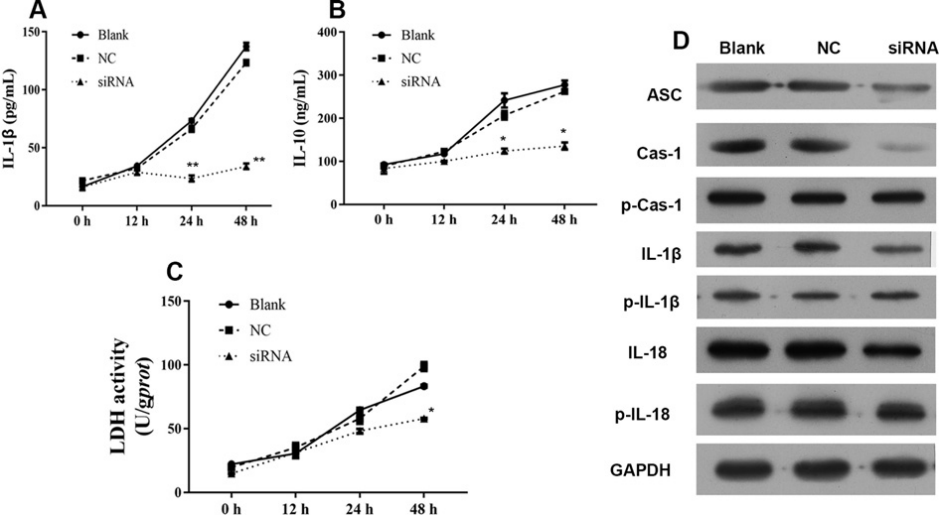
siRNA-GAS5 inhibited inflammasome formation and pyroptosis in 3AO cell line (**A**–**C**) IL-1β, IL-10, and LDH were down-regulated after treatment with siRNA–GAS5. (**D**) Expression of ASC, caspase 1, procaspase 1, IL-1β, pro-IL-1β, IL-18, and pro-IL-18 were detected using Western blot. p-Cas-1 indicates procaspase 1; p-IL-1β indicates pro-IL-1β; p-IL-18 indicates pro-IL-18. *indicates *P*<0.05, **indicates *P*<0.01.

In order to understand the mechanism of antitumor effect of lncRNA GAS5, levels of IL-1β, IL-10, and LDH were detected after transfection with siRNA for 12, 24, and 48 h. As shown in Figure 3, lncRNA GAS5 knockdown by siRNA significantly inhibited IL-1B, IL-10 levels at 24 and 48 h as well as the LDH activity at 48 h (Figure 3A–C). Further, suppressed expressions of ASC, caspase 1, IL-1β, and IL-18 were detected by Western blot (Figure 3D).

### Overexpression of lncRNA GAS5 inhibits ovarian cancer cell viability

**Figure 4:**
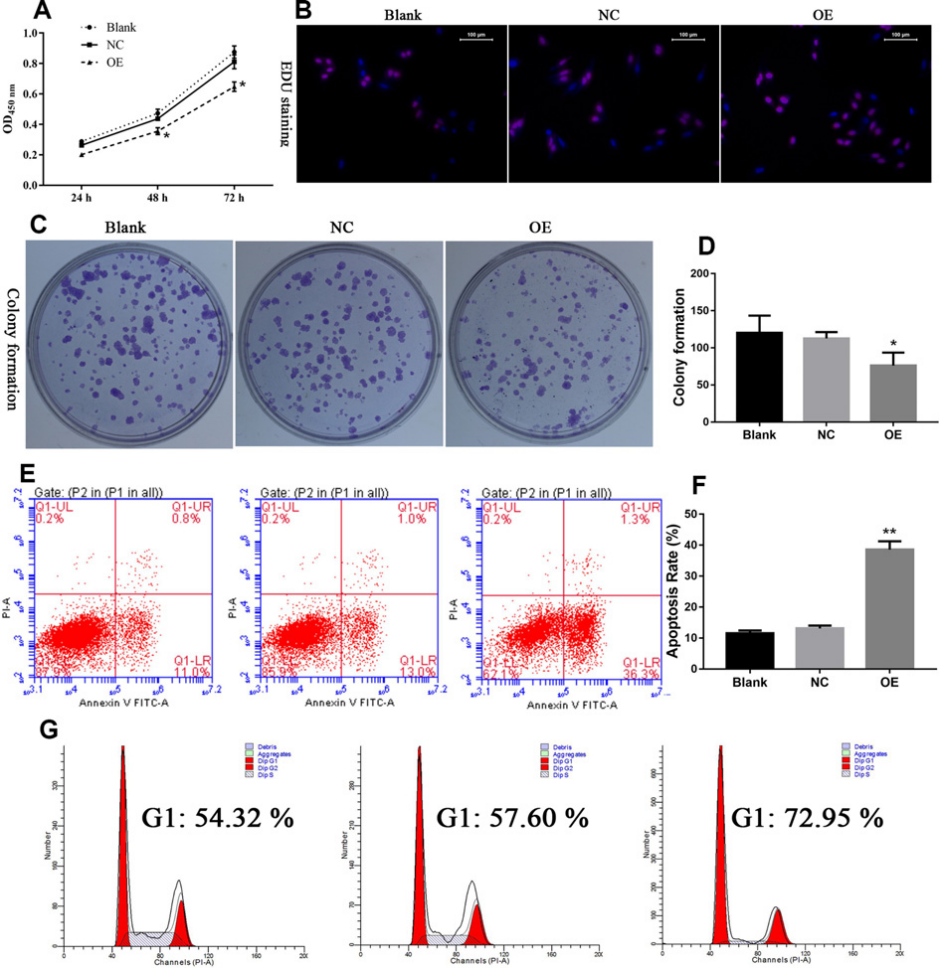
Overexpressed GAS5 inhibited ovarian cancer malignant behavior in A2780 (**A**) GAS5 hindered A2780 cell proliferation. (**B**) GAS5 inhibited EdU staining. (**C** and **D**) Colony formation was inhibited after the cells were transfected with GAS5 overexpressed plasmid. (**E** and **F**) Apoptosis rate was induced after GAS5 overexpressed. (**G**) G1 phase block was induced after cell treatment with GAS5 overexpressed plasmid; *indicates *P*<0.05, **indicates *P*<0.01.

To investigate the effect of exogenous overexpression of lncRNA GAS5 on ovarian cells, A2780 cell line, due to its relative lower level of lncRNA GAS5 expression, was transfected with GAS5 plasmid (Figure 1B). CCK-8 assay showed that cell proliferation was significantly impaired in the pCDNA3.1-GAS5-transfected A2780 cells (Figure 4A). The proportion of cells that incorporated EdU was lower in pCDNA3.1-GAS5 vector transfection as compared with pcDNA3.1 vector transfection (Figure 4B). After exogenous overexpression of GAS5, A2780 cells exhibited significantly decreased capability of colony formation as compared with mock group (Figure 4C and D).

To investigate the effect of GAS5 on cell apoptosis, Annexin V-FITC/PI staining method was used. The results showed increased apoptosis rate of A2780 cells following pCDNA3.1-GAS5-transfection as compared with pCDNA3.1 vector transfection (Figure 4E and F). Cell cycle assay was performed using Flow Cytometer. The results showed increased percentage of cells in G1 phase after GAS5 overexpression.

These results indicated that overexpression of GAS5 in A2780 could induce G1 phase block and attenuate cell proliferation.

### Overexpression of lncRNA GAS5 induces inflammasome formation and pyroptosis

**Figure 5:**
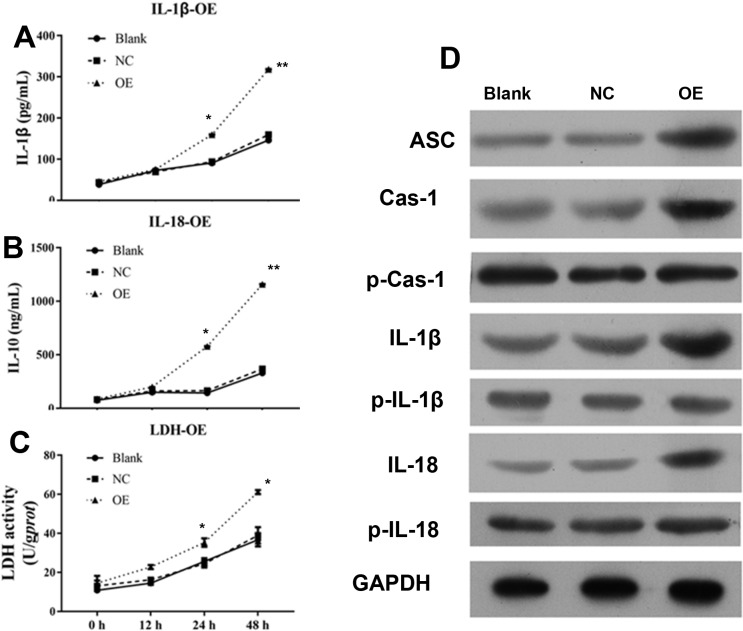
GAS promotes inflammasome formation and pyroptosis in A2780 cell line (**A**–**C**) Elevated IL-1β, IL-10, and LDH were detected by ELISA methods after treatment with siRNA–GAS5. (**D**) Expression of ASC, caspase 1, procaspase 1, IL-1β, pro-IL-1β, IL-18, and pro-IL-18 was detected using Western blot. p-Cas-1 indicates procaspase 1; p-IL-1β indicates pro-IL-1β; p-IL-18 indicates pro-IL-18; *indicates *P*<0.05, **indicates *P*<0.01.

To confirm the correlation between GAS5 expression and inflammasome, IL-1β, IL-10, LDH, and the expression of inflammasome protein were determined. As shown in Figure 5, significantly elevated IL-1β, IL-10, and LDH expression was found after cells were transfected with GAS5 overexpression plasmid for 24 and 48 h (Figure 5A–C). Further analysis showed that overexpression of lncRNA GAS5 induced a time-dependent activation of ASC, caspase-1, and IL-1β as detected by Western blot.

Our results suggested that pyroptosis in these lncRNA GAS5 overexpressed cells was induced in a time-dependent manner. Thus, these findings indicated that lncRNA GAS5 exerted its antitumor effect through lncRNA GAS5 inflammasome formation.

### Overexpression of GAS5 and induction of inflammasome formation *in vivo*

**Figure 6:**
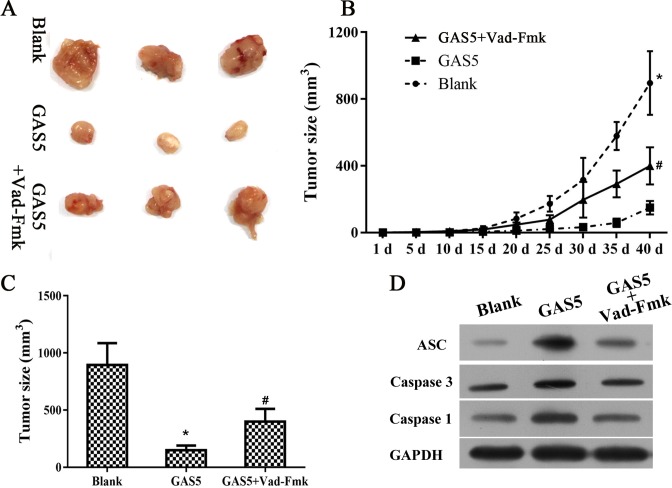
Inflammasome inhibitor, Vad-Fmk, reversed the antitumor effect *in vivo* (**A**–**C**) Vad-Fmk blocked the inhibition of GAS5 anti tumor effect. **(D**) ASC and caspase 3 were down-regulated after treatment with GAS5 and Vad-Fmk; *indicates *P*<0.05 vs Blank group, ^#^indicates *P*<0.05 vs GAS5 overexpressed group.

To investigate the GAS5 antitumor function, ovarian cancer xenograft model in nude mice was used. As shown in Figure 6A–C, GAS5 inhibited tumor development as compared with blank group; however, Vad-Fmk, an inhibitor of inflammasome reversed the inhibition of GAS5. Finally, expression of ASC and Caspase 3 protein was determined and results showed a positive correlation between ASC, a biomarker of inflammasome, and Caspase 3 (Figure 3D).

## Discussion

Accumulative evidence have showed that lncRNAs play essential roles in the development and progression of cancer and affect epigenetic information and promote cellular growth [20,21]. Preventing oncogenesis and treating cancer successfully by effectively controlling cell proliferation and survival are critical to decrease rate or mortality associated with ovarian cancer. Thus, exploring the biological functions of lncRNAs in cancer development may provide potential clue for its therapy.

LncRNA GAS5 has gained increasing attention in cancer research owing to its ubiquitously high expression during growth arrest. The abnormal down-regulation of lncRNA GAS5 has been observed in various cancers, such as breast cancer, renal cell carcinoma, bladder cancer, prostate cancer, and pancreatic cancer [10,16,17]. Moreover, various studies have demonstrated a correlation between overexpression of lncRNA GAS5 through regulation of cell cycle, cell apoptosis, and promote cell death and cellular growth inhibition of different cancer cell lines [17]. These results indicate that lncRNA GAS5 is a tumor suppresser gene. However, little is known regarding its expression in ovarian cancer.

The present study explored the role of lncRNA GAS5 in ovarian cancer. We detected the RNA expression of lncRNA GAS5 in patients diagnosed with ovarian cancer. Importantly, our results showed that expression of lncRNA GAS5 in 20 patients with ovarian cancer was lower in cancer tissues as compared with adjacent normal counterparts (Figure 1A). Knockdown and overexpression of lncRNA GAS5 were design to further study the role of lncGAS5 in ovarian cancer cells.

LncRNA GAS5 knockdown by siRNA, the ability of proliferation and colony formation of ovarian cells were significantly enhanced (Figures 2 and 3). Following exogenous overexpression of lncGAS5, cell proliferation, and colony formation ability of ovarian cells were significantly inhibited as compared with mock group (Figures 4 and 5). These findings indicated that loss of lncRNA GAS5 expression may contribute to disease progression. These functions were also observed in other cancers, such as breast cancer [18], stomach cancer [22], prostate cancer [23], and non-small-cell lung cancer [24].

Although lncRNA GAS5 was suggested to play an important role in inhibiting cancer development, the underlying mechanism of lncRNA GAS5 is still unclear. In the present study, we showed that lncRNA GAS5 plays a protective role ovarian cancer by inflammasome formation. There results were confirmed by protein expression of cleaved caspase-1. In addition, induction of pyroptosis is characterized by release of LDH, which is normally maintained within the cell cytosol. Our data showed that lncRNA GAS5 overexpression could induce pyroptosis in a time-dependent manner (Figure 5), and pyroptosis was inhibited when lncRNA GAS5 was knockdown. Overexpression of lncRNA GAS5 induced a time-dependent release of IL-1β and IL-18, as detected by ELISA, while knockdown of lncRNA GAS5 was associated with decreased concentrations of IL-1β and IL-18 in a time-dependent manner. In addition, these experiment models and animal investigations also supported the role of lncRNA GAS5 as tumor suppressor.

LncRNA GAS5 may function as a suppressor of tumor progression by inducing inflammasome formation, which, in turn, actives inflammatory processes [13], to induce cell pyroptosis, a process characterized as programmed cell death [14]. LncRNA GAS5, which accumulates in growth-arrested cells, acts as a decoy hormone response element for the glucocorticoid receptor (GR) and, hence, blocks the up-regulation of gene expression by activating GR [25,26]. The GR is expressed in every cell of the body, which regulates genes expression controlling the development, metabolism, and immune responses [27,28]. GR binds to glucocorticoids to regulate the gene transcription. The activated GR complex up-regulates the expression of anti-inflammatory proteins in the nucleus or represses the expression of proinflammatory proteins in the cytosol. Therefore, by interfering with GR, lncRNA GAS5 play a role in inflammasome formation and promote inflammatory process. Accumulated evidence have shown that inflammasome in a component of the innate immune system that is an important component in limiting cancer progression [29].

Taken together, our results indicated that lncRNA GAS5 could inhibit tumor progression by promoting ovarian cancer cell apoptosis and pyroptosis. Decreased expression of GAS5 could lead to ovarian cancer development. However, the underlying mechanism of lncRNA GAS5 in regulating the target expression in ovarian cancer cells needs further study.

## Conclusion

Our study showed that lncRNA GAS5 was decreased in human ovarian cancer tissues, thereby indicating that GAS5 may play a role as tumor suppressor and an indicator of poor prognosis in ovarian cancer patients. A better understanding of the mechanism of GAS5 in the evolution and progression of human ovarian cancer may lead to new diagnostic and therapeutic approaches for ovarian cancer.

## Abbreviations

ASC: Apoptosis associated speck-like protein containing CARD
GAS5: growth arrest-specific transcript 5
GR: glucocorticoid receptor
lncRNA GAS5: long noncoding RNA growth arrest-specific transcript 5
