# Expression of Concern: LncRNA GAS5 suppresses ovarian cancer by inducing inflammasome formation

**DOI:** 10.1042/BSR-2017-1150_EOC

**Published:** 2023-02-09

**Keywords:** apoptosis, cell proliferation, growth arrest-specific 5, inflammasome, long non-coding RNA, ovarian cancer

The Editorial Office has been made aware of potential issues surrounding the scientific validity of this paper, hence has issued an expression of concern to notify readers whilst the Editorial Office investigates. There appears to be a duplicated image, with some possible signs of alteration, between the first two panels of Figure 4E.

